# Associations of physical activity with phase angle in adolescents living with HIV: The moderating and mediating roles of physical fitness

**DOI:** 10.14814/phy2.70696

**Published:** 2026-02-03

**Authors:** Caio César da Silva Moura Santos, Christefany Régia Braz Costa, Gustavo Gomes de Araujo, Diego Augusto Santos Silva, Analiza Mónica Silva, Luiz Rodrigo Augustemak de Lima

**Affiliations:** ^1^ Laboratory of Research in Biodynamics of Human Performance and Health – LAPEBIOS Maceió Brazil; ^2^ Graduate Program in Health Sciences Federal University of Alagoas Maceió Brazil; ^3^ Graduate Program in Nursing Federal University of Alagoas Maceió Brazil; ^4^ Graduate Program in Physical Education Federal University of Santa Catarina Florianópolis Brazil; ^5^ Exercise and Health Laboratory, CIPER, Faculdade Motricidade, Humana Universidade de Lisboa Lisbon Portugal; ^6^ Department of Movement Sciences and Sports, Training, School of Sport Sciences The University of Jordan Amman Jordan

**Keywords:** adolescents, HIV, mediating, moderating, physical activity, physical fitness

## Abstract

HIV infection and combination antiretroviral therapy (cART) can cause metabolic and cardiovascular changes in adolescents, who often have low physical activity (PA), harming their health. To investigate the relationship between PA levels and phase angle (PhA), we analyze potential moderating and mediating effects. Cross‐sectional study with 47 adolescents (10–18 years) with vertically transmitted HIV. PA was assessed using PAQ‐C; PhA was measured by tetrapolar bioelectrical impedance. Aerobic capacity was assessed by a submaximal bench step test, muscular strength by handgrip test, and body composition by anthropometric measures (arm muscle area [AMA] and body fat percentage [%BF]). Correlation, regression, and mediation and moderation analyses were performed. 61.7% showed inadequate PhA (<5.0°), mostly girls. A significant correlation existed between PA and PhA (*r* = 0.39; *p* = 0.01), maintained in adjusted regressions (*β* = 1.087; *p* = 0.001). General mediation and moderation effects were not confirmed; however, conditional analyses revealed high muscular strength significantly moderated the PA–PhA link (*β* = 1.0537; *p* = 0.0024). VO_2 peak_, %BF, and AMA showed significant conditional effects at different levels. PA and PhA are directly associated, independent of confounders, and muscular strength, aerobic capacity, and body composition partially moderate this relation in adolescents with HIV.

## Graphical abstract

The graphical abstract illustrates the association between physical activity levels and cellular health in adolescents living with HIV (ALHIV). Cellular health was assessed using phase angle (PhA), derived from bioelectrical impedance analysis, a marker of cell membrane integrity and body cell mass. Higher physical activity levels were positively associated with PhA, independent of age, sex, viral load, and antiretroviral therapy (ART). Although mediation models were not supported, aerobic fitness (VO_2 peak_) and body fat percentage partially moderated this relationship, indicating that the magnitude of the association varied according to physical fitness and body composition. A high prevalence of physical inactivity and inadequate PhA values was observed, underscoring the vulnerability of this population and supporting the relevance of physical activity and body composition management as components of care for adolescents living with HIV.

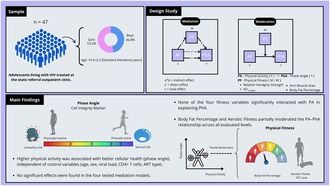

## 1 INTRODUCTION

In 2022, an estimated 77,000 new HIV infections occurred among people aged 15–19 years, increasing the global number of adolescents living with HIV (ALHIV) to approximately 690,000 (UNICEF, 2023). Most acquire HIV through vertical transmission and live with chronic exposure to both the virus and antiretroviral therapy (ART) (Adamson, 2012; Barlow‐Mosha et al., 2013). These exposures can lead to cardiometabolic (Amato et al., 2013; Fernström et al., 2017; Soysal et al., 2021), such as dyslipidemia, insulin resistance, and lipodystrophy, which are linked to reduced physical fitness and altered body composition (Barlow‐Mosha et al., 2013; Carr et al., 1998; Medeiros et al., 2021).

Children and adolescents with chronic conditions face limitations in participating in physical activities (PA) and sports due to both physical and physiological restrictions, as well as perceptions imposed by their disease (van Brussel et al., 2011). Research has rarely explored physical fitness components such as muscular strength and endurance, cardiorespiratory fitness, and flexibility in adolescents with HIV, with most studies focusing on body composition (de Castro et al., 2024).

There is evidence that ALHIV exhibit reduced muscular strength, impaired body composition (characterized by high body fat and low fat‐free mass), and low levels of habitual PA (Chirindza et al., 2022). Furthermore, aerobic fitness and PA (in continuous blocks of 5 and 10 min at moderate‐to‐vigorous intensity) were lower than those of their healthy peers (de Lima, Silva, et al., 2017). A systematic review suggested a high prevalence of physical inactivity among Brazilian ALHIV (da Silva & Andaki, 2020). A higher level of weekly PA can improve the overall clinical condition of individuals with chronic conditions, including ALHIV (Bull et al., 2020; de Lima et al., 2019; Martins et al., 2017).

In this context, assessing sensitive biomarkers that reflect both physical fitness and underlying health status becomes essential. One such indicator is the phase angle (PhA), which has gained attention for its prognostic value in clinical populations (Kyle et al., 2004). Evidence suggests that PhA is a reliable marker of cell membrane integrity and overall cellular mass (Martins et al., 2023). PhA is defined as the ratio between resistance (i.e., representing body fluids) and reactance (i.e., reflecting the capacitance of cell membranes), both parameters measured through bioelectrical impedance analysis (Kyle, 2004). PhA has shown prognostic value in assessing outcomes in non‐communicable chronic diseases, as lower values have been associated with early mortality (Garlini et al., 2019; Schwenk et al., 2000).

PhA in ALHIV has been positively associated with aerobic and muscular fitness across various chronic conditions and age groups, including adolescents (Martins et al., 2022). Other components of body composition, such as fat‐free mass, have shown a direct association with PhA, while no consensus exists regarding its relationship with fat mass (Martins et al., 2023). All these components of physical fitness may influence the relationship between PA levels and PhA. Likewise, the relationship between PA and PhA may be altered under different conditions of physical fitness, considering that physiological variations determine aerobic and strength performance, as well as morphological variations in body fat and muscle mass.

Although there is evidence of a relationship between physical fitness parameters and PhA, a direct association between PA and PhA has only been observed in healthy individuals and those with chronic conditions across different age groups—with the exception of adolescence. Thus, there is a gap in the literature regarding the investigation of the relationship between PA levels and PhA, especially in ALHIV, with no existing evidence to date of studies that have tested models considering the mediating or moderating effects of physical fitness in this relationship.

Given this gap, the present study aims to investigate the association between PA and PhA in ALHIV, as well as to examine the potential mediating and moderating roles of physical fitness in this relationship. This study was guided by the following theoretical hypotheses: (1) there is a positive and significant association between PA levels and PhA values in ALHIV; (2) physical fitness mediates the relationship between PA and PhA, such that higher levels of PA are associated with better physical fitness, which in turn is associated with higher PhA values; and (3) physical fitness also moderates the relationship between PA and PhA, such that the effect of PA on PhA varies depending on physical fitness levels, with stronger effects observed among adolescents with higher fitness levels. By explicitly stating these hypotheses, the study seeks to clarify its theoretical framework and provide a foundation for understanding the potential roles of physical fitness in the relationship between physical activity and PhA in ALHIV.

## 2 METHOD

The methods of this study were structured according to the STROBE– Strengthening the Reporting of Observational Studies in Epidemiology checklist, as detailed in Appendix S1.

### 2.1 Study design

This was a single‐center, cross‐sectional observational study conducted from March 2022 to December 2023 in the city of Maceió, Alagoas, located in northeastern Brazil. All assessments were carried out at the Specialized Care Service (SCS) of Dr. Hélvio Auto Hospital (HEHA), a referral center for pediatric HIV care in the state of Alagoas, under the Brazilian Unified Health System (SUS). The region has a Human Development Index (HDI) of 0.684, which has declined by 0.44% since 2019 and remains below the national Brazilian HDI of 0.754 (Atlas Brasil, 2022; IBGE, 2022). This suggests that the social vulnerability of the population served at HEHA is both present and increasing.

The exposure variable was PA, and the outcome was the PhA. Moderating and mediating variables included aerobic fitness, muscle strength, AMA, and body fat percentage (%BF). Covariates were sex, age, CD4+ T lymphocyte count, viral load, sexual maturation stage, and ART. This study is a secondary analysis of data from the umbrella project “Saúde PositHIVa do Adolescente Alagoano: Monitoramento do Estilo de Vida, Aptidão Física, Cognição e Risco Cardiometabólico”, funded through the FAPEAL Call 06/2020 – PPSUS (Programa Pesquisa para o SUS: Gestão Compartilhada em Saúde), supported by the Department of Science and Technology of the Ministry of Health (Decit‐SCTIE‐MS), the Brazilian National Council for Scientific and Technological Development (CNPq), the Alagoas State Research Foundation (FAPEAL), and the Alagoas State Health Secretariat (SESAU‐AL).

### 2.2 Ethical considerations

This study was approved by the Human Research Ethics Committee (HREC) of the State University of Health Sciences of Alagoas (approval no. 4.564.290) and by the HREC of the Federal University of Alagoas (approval no. 4.506.466). All procedures were conducted in accordance with the ethical standards of the institutional research committees and with the principles outlined in the Declaration of Helsinki. Written assent was obtained from all participants, and informed consent was provided by their respective legal guardians.

### 2.3 Population and sample

A total of 72 ALHIV were receiving care at the SCS at the HEHA in 2022. Post hoc sample size calculation was performed using G*Power software (version 3.1.9.7) to determine the appropriate sample size for multiple linear regression analyses. Considering a medium effect size (*f* ^2^ = 0.15), a significance level of 5% (*α* = 0.05), and a statistical power of 80% (1‐*β* = 0.80), with one predictor (PA level) included in the analysis, the calculation indicated a required sample size of 55 participants. (Cohenn, 1988).

**FIGURE 1:**
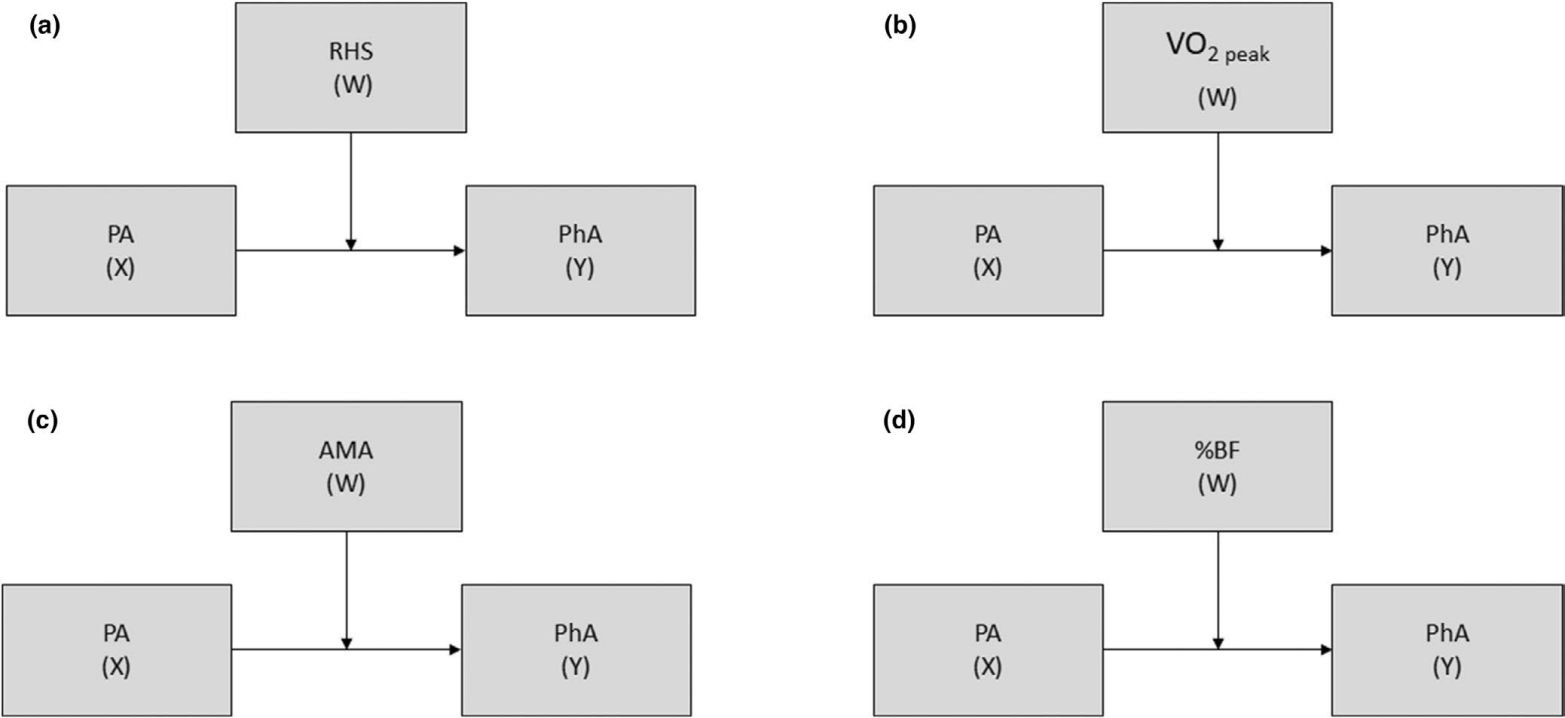
Four simple moderation models tested. %BF, body fat percentage; AMA, arm muscle area; PA, physical activity; PhA, phase angle; RHS, relative handgrip strength; VO_2_ peak, peak oxygen uptake; W, moderating variable; X, independent variable; Y, dependent variable.

In 2022, a total of 72 adolescents living with HIV were receiving care in the state. Fifty‐nine were assessed. Three were excluded: one due to pregnancy and two due to physical disabilities that prevented participation in physical assessments. Moreover, nine participants were excluded due to missing data on ART in their medical records. This missing data was related to inconsistencies in the national health registry (SUS), where personal records had not been properly updated by healthcare services. As a result, ART information was either outdated or linked to duplicated identities in the system—meaning the accessible records reflected old data, while updated information was associated with a new identification number that could not be accessed through the electronic medical system. Therefore, the final sample included in the analysis consisted of 47 participants (see Figure 1).

The eligibility criteria for participation in this study were as follows: (1) adolescents aged 10–18 years with a confirmed diagnosis of HIV acquired through vertical transmission; (2) currently receiving follow‐up care at the SAE‐HEHA; and (3) having sufficient physical and cognitive capacity to participate in the study procedures, including the ability to walk and perform physical exercise. The exclusion criteria were as follows: to obtain medical contraindications preventing engagement in physical exercise.

### 2.4 Independent variable

Habitual PA was assessed using the Physical Activity Questionnaire for Older Children (PAQ‐C) (Crocker et al., 1997). This questionnaire has been validated, and its score (ranging from 1 to 5) was significantly correlated with moderate‐to‐vigorous PA levels measured by accelerometry in ALHIV (*r* = 0.506, *p* < 0.001) (de Castro et al., 2023).

The PAQ‐C consists of nine structured questions designed to capture various aspects of PA over the past 7 days. These include items on the weekly frequency of PA during leisure time; participation in sports and games; PA performed at school (during physical education classes and recess); and activity during different leisure periods (lunch, immediately after school, evening, and weekends). Responses are coded using a 5‐point Likert scale. The final score is calculated as the arithmetic mean of the nine items (Crocker et al., 1997). Lower scores (<2.15) indicate insufficient levels of PA, while higher scores (≥2.15) classify individuals as physically active. These cutoff points were established in a study that simultaneously employed accelerometry and used the benchmark of 60 min of moderate‐to‐vigorous PA per day for children and ALHIV (de Castro et al., 2023).

### 2.5 Dependent variable

The dependent variable was PhA, assessed using bioelectrical impedance analysis (BIA) with a tetrapolar device model BIA1010 (Sanny®, São Bernardo do Campo, BR). The device was inspected and calibrated by the manufacturer prior to data collection, and calibration was also performed by the lead researcher before evaluations, following the manufacturer's instructions. Reproducibility data for this device reported in the literature indicate a coefficient of variation (CV) of 0.17% for resistance (R) and 0.72% for reactance (Xc), while the technical error of measurement was 0.76 Ω (0.22%) for R and 0.35 Ω (0.92%) for Xc (Costa et al., 2022).

Standard conditions for optimal BIA measurement were followed, including removal of metallic objects, confirmation of fasting status, avoidance of coffee or diuretics, screening for pacemakers or metallic implants, and bladder emptying if necessary; participants underwent brief clinical interviews based on established criteria (Kyle et al., 2004). For standardization, sensing electrodes were placed on the dorsal wrist and ankle, while source electrodes were positioned at the base of the metacarpophalangeal joints (third metacarpal and third metatarsophalangeal regions); measurements were taken on the right side of the body with adolescents lying supine on a non‐conductive surface, skin cleansed at electrode sites, arms angled 30°–45° from the torso, and legs similarly spaced.

The PhA was obtained through the R and the Xc, it is obtained through the BIA, at a frequency of 50 kHz. Calculated by $\mathrm{PhA}=\mathrm{Xc}/R\times \left(180^{{}^{\circ}}\right)/\pi$ (Norman et al., 2012). PhA values range from 2.0° to 9.5°. Values below 5.0° was considerate inadequate, indicating poor cellular health and a higher risk of early mortality, whereas values above 5.0° can be interpreted as indicative of better health status and lower mortality risk (Martins et al., 2020; Norman et al., 2012).

### 2.6 Mediator and moderator variables

Handgrip strength was assessed using a hydraulic dynamometer (model SH5001, Saehan©, Masan, South Korea). The Saehan dynamometer has shown high agreement with the Jamar dynamometer (*r* = 0.985), which is widely used in population‐based studies (Bohannon et al., 2006; Gómez‐Campos et al., 2018; Reis & Arantes, 2011). Maximum isometric strength was measured in both hands, and the average value (in kilograms) was subsequently calculated. Classification as “adequate” or “inadequate” strength was based on the 20th percentile of the average grip strength distribution (Marmol‐Perez et al., 2024), using reference values from a population‐based study conducted with European children and adolescents (Ortega et al., 2023), using sex‐ and age‐specific cutoff points.

Cardiorespiratory fitness was assessed using the Modified Canadian Aerobic Fitness Test (MCAFT Step Test) (Lang et al., 2020), with heart rate monitored via a chest strap heart rate sensor (Polar H10, Polar®, Finland) placed at the xiphoid process, located at the lower part of the sternum. The protocol followed a submaximal approach, with the test being terminated once the participant reached 85% of their age‐predicted maximum heart rate, estimated using the Equation (220 – age), as suggested in the MCAFT protocol (Weller et al., 1993). Peak oxygen uptake (VO_2 peak_) was estimated from the following equation: 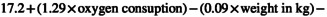 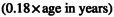. VO_2 peak_ was classified as either adequate or inadequate based on Z‐score cut‐off points associated with cardiometabolic risk in adolescents, taking into account the participant's age and sex (Lang et al., 2020).

VO_2 peak_ was classified as “adequate” for females aged 9–13 years when values exceeded 46.4 mL/kg/min and for males when values exceeded 48.7 mL/kg/min. Values below these thresholds were considered “inadequate.” For adolescents aged 14–18 years, VO_2 peak_ values above 36.5 mL/kg/min for females and above 46.1 mL/kg/min for males were considered “adequate,” while lower values were classified as “inadequate” (Lang et al., 2020).

Anthropometric measurements included body mass (kg), assessed using a Tanita® electronic scale (BF683Q, Arlington Heights, USA); height (cm), measured with a portable stadiometer (Cescorf®, Porto Alegre, RS, Brazil); and arm circumference (cm), measured using a non‐elastic anthropometric tape (Cescorf®, Porto Alegre, RS, Brazil). Skinfold thicknesses at the triceps, subscapular, abdominal, and calf sites were measured using a Lange® skinfold caliper (Beta Technology Inc., Santa Cruz, USA) to estimate body composition variables.

%BF was calculated using the equation: 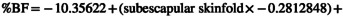 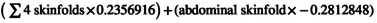 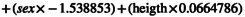 which was previously developed and validated against a gold‐standard method, demonstrating a high predictive value (*R* ^2^ = 0.85) for ALHIV (de Lima, Martins, et al., 2017). %BF classifications considered “below ideal” and “ideal” ranges as a single category to create a dichotomous variable (“below/ideal” vs. “above”). For males, the ideal range was defined as 10%–20% body fat, and for females, 15%–25% (Lohman, 1992). Furthermore, to assess the muscle mass component, the Arm Muscle Area (AMA) was calculated from the arm perimeter and triceps skinfold measurements applied in the formula: $\mathrm{AMA}\;\left({\mathrm{cm}}^{2}\right)=\left(\left(\mathrm{Arm}\;\text{perimeter}-\pi \;\text{triceps skinfold}\;\right)2\;\right)/\left(4\;\pi \right)$. Arm muscle area was classified as adequate or inadequate using the 25th percentile as the cutoff point, considering age and sex, based on data from the National Health and Nutrition Examination Survey (NHANES) (Frisancho, 1990).

### 2.7 Covariates

Age and sex were self‐reported. CD4+ T lymphocyte count was categorized into three groups: severe immunosuppression (<15%), evidence of moderate immunosuppression (15%–24%), and no evidence of immunosuppression (≥25%) (Centers for Disease Control (CDC), 1987). Sexual maturation stage was self‐reported. In a private setting, a researcher of the same sex as the adolescent provided a detailed explanation of the Tanner scale (Sociedade Brasileira de Pediatria, n.d.), after a detailed description of the participant's genitals, self‐reported and offered by a researcher of the same sex in a private environment. Sociodemographic data were obtained through a questionnaire, and data regarding viral load, CD4+ T lymphocyte count, type of ART (ART combination table available in Table S1), and time on ART were obtained from medical records.

### 2.8 Statistical analysis

Data were tabulated using Google Sheets®. All statistical analyses were conducted using STATA® for Windows version 13.0, except for mediation and moderation analyses, which were performed in IBM SPSS® Statistics version 22.0 using the PROCESS macro (Hayes, 2022). A significance level of *p* < 0.05 was adopted for all analyses.

Descriptive statistics (absolute and relative frequencies), measures of central tendency (mean and median), and measures of dispersion (interquartile range and standard deviation) were calculated. The Shapiro–Wilk test was used to assess the normality of data distribution, along with histogram analysis and evaluation of skewness and kurtosis (values greater than 3 or less than −3 were considered indicative of non‐normal distributions). Linear correlation analyses (Pearson or Spearman) were conducted to explore bivariate relationships between continuous variables and the chi‐square or Fisher's exact test to test categorical associations. A multiple linear regression model was performed to test the relationship between the level of PA (independent variable) and PhA (dependent variable), adjusted for covariates (age, sex, sexual maturation, viral load, CD4+ T lymphocytes, and type of ART).

Moderation and mediation analyses were performed using the mediating and moderating variables to compose four simple mediation (see Figure S1) and moderation (see Figure 1) models (Bolin, 2014): PA level (independent variable [X]), physical fitness (RHS, relative handgrip strength; VO_2 peak_, peak oxygen uptake; AMA, arm muscle area, and %BF) (mediator variables [M] and moderators [W]), PhA (dependent variable [Y]), and adjustment variables (Sexual, Viral load, CD4+ T lymphocytes, and type of ART).

Subsequently, moderation analyses were conducted to investigate the extent to which components of physical fitness moderated the relationship between PA level and PhA. To better interpret the effect, the moderator variable was segmented into three levels (conditional effects), using the following cutoff points: lower 16%, middle 68%, and upper 16% (Hayes, 2022).

## 3 RESULTS

**FIGURE 2:**
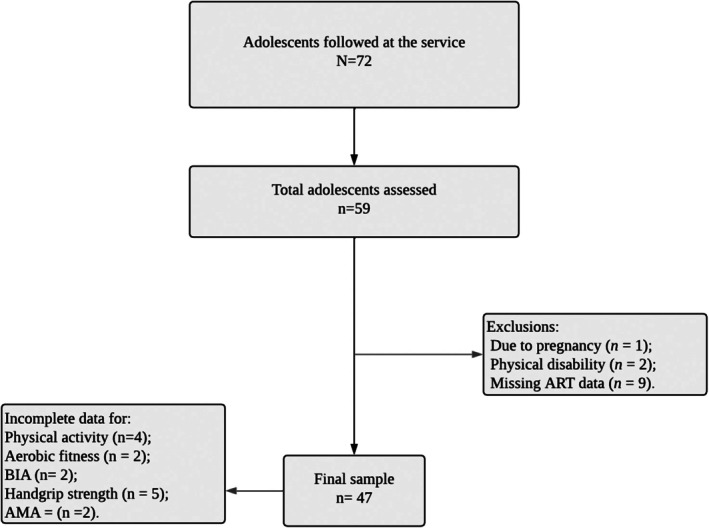
Flowchart of research data collection. Brazil. 2025. AMA, arm muscle area; ART, antiretroviral therapy; BIA, bioelectrical impedance analysis.

The present study included 47 participants (Figure 2), representing 65.28% of all eligible individuals. The mean age was 14.4 ± 2.2 years. Table 1 presents comparisons of means and medians between sexes for quantitative variables. Significant differences between males and females were observed for bioelectrical impedance resistance, relative strength, VO_2_ peak, and arm muscle area.

**TABLE 1:** Continuous parameters of characteristics of adolescents living with HIV.

Variables	Total	Girls	Boys	t/P	*p* Value
	(*n* = 47)	(*n* = 25)	(*n* = 22)		
	Mean (standard deviation) or median (interquartile range)				
Age (year)	14.4 (2.2)^a^	14.4 (2.3)^a^	14.4 (2.2)^a^	0.0547	0.9567
Weight (kg)	49.1 (40.4; 56.4)^b^	43.8 (37.4; 51.9)^b^	45.6 (37.4; 53.0)^b^	−1.087	0.2768
Height (cm)	160.0 (11.4)^a^	154.3 (8.9)^a^	156.9 (10.4)^a^	−1.8914	0.0650
Viral load (log)	3.7 (3.7; 5.6)^b^	3.7 (3.7; 8.1)^b^	3.7 (3.7; 3.7)^b^	1.353	0.1791
CD4+ t lymphocytes (%)	29.3 (10.1)^a^	31.0 (9.2)^a^	30.2 (9.6)^a^	0.975	0.5532
Time on ART (year)	9.8 (2.9)^a^	8.6 (3.4)^a^	9.2 (3.2)^a^	−1.503	0.1328
Physical activity (score)	1.6 (1.3; 2.7)^b^	1.6 (1.1; 2.2)^b^	1.6 (1.24; 2.4)^b^	−0.979	0.3277
RHS (strength/kg)	0.6 (0.6; 0.7)^b^	0.5 (0.5; 0.6)^b^	0.6 (0.5; 0.7)^b^	−2.629	**0.0086**
Sum of strength (kg)	54.0 (47.5; 75.0)^b^	48.0 (26.0; 55.0)^b^	51.0 (40.0; 60.0)^b^	−2.365	**0.0189**
VO_2 peak_ (mLO_2_/min)	52.6 (48.0; 56.4)^b^	43.7 (39.4; 49.2)^b^	48, 0 (41.3; 54.6)^b^	−3.298	**0.0010**
Arm muscle area (cm^2^)	20.4 (2.6)^a^	18.3 (1.9)^a^	19.3 (2.5)^a^	−3.1072	**0.0033**
Body fat (%)	10.9 (8.3; 18.8)^b^	14.0 (11.5; 18.5)^b^	12.6 (9.7; 18.4)^b^	1.862	0.0626
Resistance (Ω/m)	625.2 (86.0)^a^	726.8 (76.4)^a^	677.1 (95.4)^a^	4.1933	**0.0001**
Reactance (Ω/m)	52.4 (50.2; 60.4)^b^	55.1 (46.0; 66.7)^b^	52.4 (49.2; 60.5)^b^	0.352	0.7249
Phase angle (degrees)	5.1 (4.1; 5.7)^b^	4.4 (3.6; 5.0)^b^	4.7 (4.05; 5.3)^b^	−1.703	0.0886

*Note*: Bold values indicate significant *p* values.

Abbreviations: AMA, arm muscle area; ART, antiretroviral therapy; RHS, relative handgrip strength; t, critical value of the *t*‐test; U, critical value of the Mann–Whitney U test; VO_2 peak_, peak oxygen uptake.

^a^ Standard deviation.

^b^ Interquartile range.

Table 2 shows that the majority of participants (71.74%) were classified as physically inactive. A higher prevalence of participants with adequate muscle strength and peak VO_2_ was observed (87.80% and 68.96%, respectively), while all presented with inadequate AMA. In addition, most participants also had an inadequate PhA (61.70%). Regarding the frequency of types of physical activities practiced during the week by adolescents, among females, the activities more practiced every day were dancing (16.0%), walking (12.0%), and jumping (8.0%). Among males, the most practiced physical activities were cycling (18.2%), soccer/futsal (13.6%), and walking (13.6%) (Figure S2).

**TABLE 2:** Categorical characterization of adolescents with HIV.

	Total	Girls	Boys	X^2^	*p*
	(*n* = 47)	(*n* = 25)	(*n* = 22)		
	*n* (%)	*n* (%)	*n* (%)		
Skin color					
White	10 (21.74)	4 (8.69)	6 (13.04)	0.7589	0.484
Non‐white	36 (78.26)	20 (43.49)	16 (34.78)		
Income					
≤2 minimum wages	37 (80.43)	21 (45.65)	16 (34.78)	0.4423	0.711
>2 minimum wages	9 (19.57)	4 (8.69)	5 (10.87)		
Physical activity					
Active (scores ≥2.15)	13 (28.26)	6 (25.00)	7 (31.82)	0.2632	0.608
Inactive (scores <2.15)	33 (71.74)	18 (75.00)	15 (68.18)		
VO_2 peak_					
Adequate	31 (68.96)	17 (36.17)	14 (29.79)	0.0992	0.753
Inadequate	16 (34.04)	8 (17.02)	8 (17.02)		
Muscle strength					
Adequate	36 (87.80)	19 (40.34)	17 (41.46)	0.2869	0.663
Inadequate	5 (12.20)	2 (4.88)	3 (7.32)		
Body fat (%)					
Below and ideal	41 (87.23)	23 (92.00)	18 (81.82)	1.0894	0.398
Above	6 (12.77)	2 (8.00)	4 (18.18)		
Arm muscle area					
Adequate	0 (0.00)	0 (0.00)	0 (0.00)	—	—
Inadequate	45 (100.0)	23 (100.0)	22 (100.0)		
ART regjmen					
Without protease inhibitors	19 (40.43)	11 (44.00)	8 (36.36)	0.2833	0.595
With protease inhibitor	28 (59.57)	14 (56.00)	14 (56.00)		
Carga viral					
Undetectable	32 (68.09)	17 (77.27)	15 (60.00)	1.6067	0.205
Detectable	15 (31.91)	5 (22.73)	10 (40.00)		
CD4+ T Lymphocytes					
Severe immunosuppression (<15%)	2 (4.35)	2 (8.33)	0 (0)	6.0495	**0.041**
Moderate immunosuppression (15%–24%)	12 (26.09)	3 (12.50)	9 (40.91)		
Not immunosuppressed (≥25%)	32 (69.57)	19 (79.17)	13 (59.09)		
Phase angle					
Adequate (≥5.0°)	18 (38.30)	6 (24.00)	12 (54.55)	4.6204	**0.032**
Inadequate (<5.0°)	29 (61.70)	19 (76.00)	10 (45.45)		

*Note*: Minimum wage in Brazil in 2022: BRL 1212.00 per month. Bold values indicate significant *p* values.

Abbreviations: ART, antiretroviral therapy; VO_2 peak_, peak oxygen uptake; X^2^, critical value of the chi‐square.

Linear correlation analyses indicated that PhA was positively associated with PA level (*r* = 0.39; *p* = 0.01), as well as with age and viral load in sex‐stratified analyses (see Table S2). In multivariate models, significant positive associations were found between PA level and PhA in both unadjusted and adjusted models (Unadjusted: *β* = 0.791; *p* = 0.005; Adjusted: *β* = 1.087; *p* = 0.001). When stratified by sex, significant associations remained in the adjusted models (females: *β* = 0.645; *p* = 0.022; males: β = 1.627; *p* = 0.005). In the unadjusted models, a significant association between PA and PhA was observed only among males (*β* = 1.160; *p* = 0.015) (see Table 3).

**TABLE 3:** Simple and multivariate regression analysis of physical activity (independent variable) and phase angle (dependent variable) in adolescents living with HIV.

	*β* (CI 95%)	β*	*F* Value	*R* ^2^_adjusted_	*p*
Total					
Physical activity (score)	0.791 (0.257; 1.324)	0.419	8.95	0.1561	**0.005**
Adjusted model 1	1.087 (0.489; 1.685)	0.579	3.25	0.2474	**0.001**
Girls					
Physical activity (score)	0.374 (−0.225; 0.974)	0.280	1.70	0.0323	0.207
Adjusted model 2	0.645 (0.110; 1.180)	0.524	2.77	0.3174	**0.022**
Boys					
Physical activity (score)	1.160 (0.249; 2.071)	0.511	7.06	0.2239	**0.015**
Adjusted model 2	1.627 (0.562; 2.692)	0.716	3.51	0.3740	**0.005**

*Note*: Adjusted model 1—Sex, age, sexual maturation, viral load, CD4+ T lymphocytes and type of ART; Adjusted model 2—Age, sexual maturation, viral load, CD4+ T lymphocytes and type of ART. β*, standardized beta coefficient. Bold values indicate significant *p* values.

As shown in Figure S3, no significant results were observed in the four mediation models tested. That is, although there was a significant association between X (physical activity) and Y (phase angle) in both the direct and total effects, none of the models met the criteria for mediation as proposed by Andrew Hayes (Bolin, 2014), because the indirect effects (X → M and M → Y) of the four mediation models were not significant.

**FIGURE 3:**
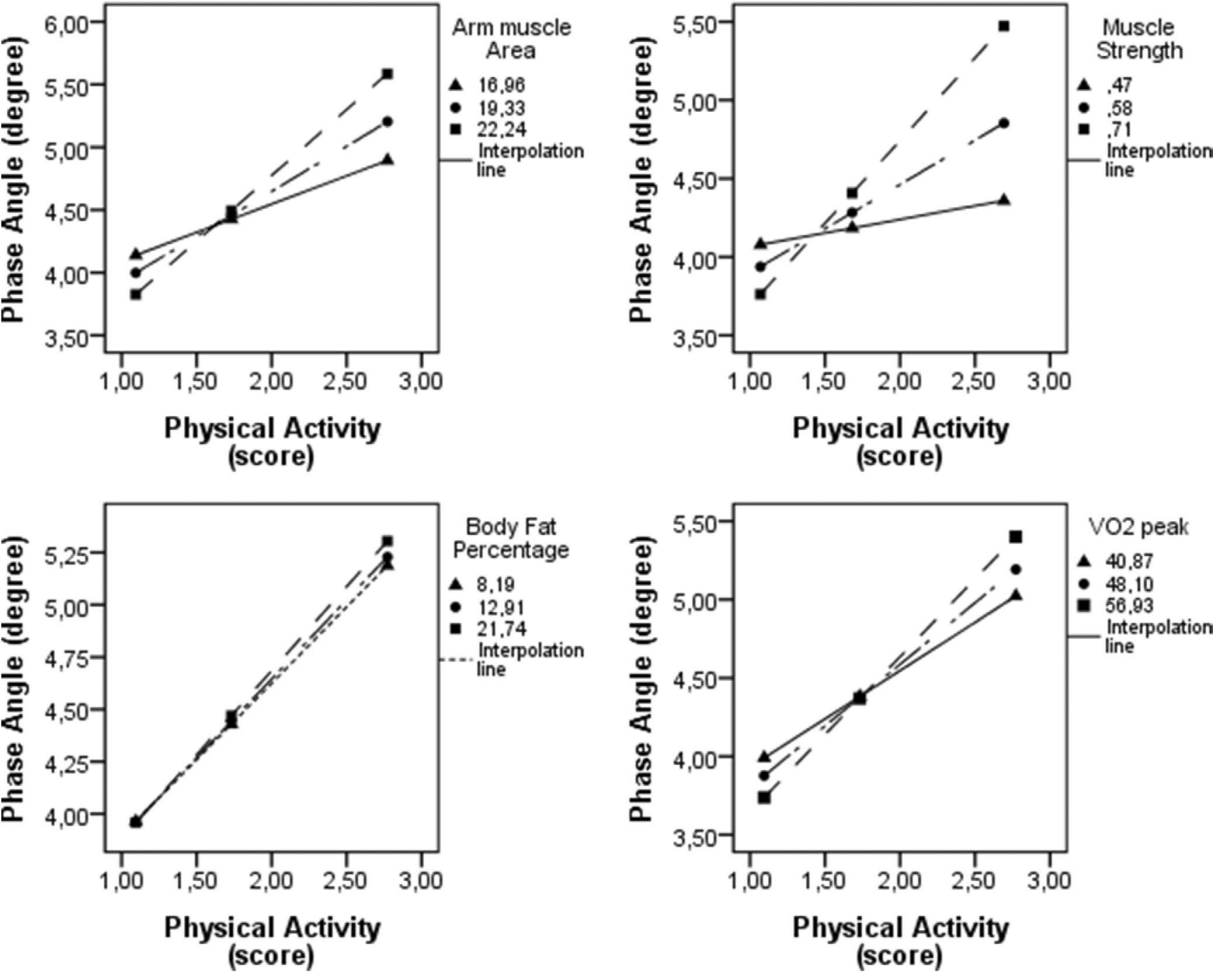
Graphical representation of the moderating effects of physical fitness variables on the relationship between physical activity and phase angle in adolescents living with HIV. VO_2_ peak, peak oxygen uptake.

Table 4 shows the moderation analyses. The physical fitness variables (W) do not appear to significantly influence the results in combination with the level of PA (X), as indicated by the X*W values. However, significant conditional effects were observed (Table 4) and are summarized in Figure 3. The %BF in very low (*β* = 0.7304; *p* = 0.0391), intermediate (*β* = 0.7557; *p* = 0.0073), and high (*β* = 0.8032; *p* = 0.0229) values showed a significant effect on the relationship between PA and PhA. The AMA showed a significant effect in intermediate (*β* = 0.7186; *p* = 0.0065) and high (*β* = 1.0488; *p* = 0.0052) values. Muscle strength at high values (*β* = 1.0537; *p* = 0.0024) showed a significant effect on the relationship between PA level and PhA. VO₂ peak also showed a statistically significant effect in low (β = 0.6153; *p* = 0.0428), intermediate (*β* = 0.7847; *p* = 0.0022), and high (*β* = 0.9913; *p* = 0.0039) conditions.

**TABLE 4:** Effects of physical fitness moderation models (body composition, muscular, and aerobic fitness) on the relationship between physical activity level and phase angle adjusted for age, sex, sexual maturation, viral load, CD4+ T lymphocytes, and type of ART. Adolescents diagnosed with HIV+.

	Coefficient	Standard error	T	*R* ^2^	*p*		Coefficient	Standard error	T	*R* ^2^	*p*
%BF						Muscle Strength					
Constant	0.9722	1.7486	0.5560	0.4885	0.5823	Constant	5.3340	3.0371	1.7563	0.7429	0.0904
PA (X)	0.6863	0.5540	1.2389		0.2250	PA (X)	−1.5068	1.3622	−1.1062		0.2784
BFM (W)	−0.0063	0.0650	−0.0972		0.9232	Muscle Strength (W)	−5.1189	3.5232	−1.4529		0.1578
PA*%BF (X*W)	0.0054	0.0327	0.1642		0.8707	PA*Muscle Strength (X*W)	3.5825	2.1204	1.6896		0.1026
Conditional effects—%BF (W)						Conditional effects—Muscle Strength (W)					
8.1885 (16% lower)	0.7304	0.3385	2.1577		**0.0391**	0.4690 (16% lower)	0.1734	0.4298	0.4035		0.6898
12.9077 (64% middle)	0.7557	0.2628	2.8760		**0.0073**	0.5780 (64% middle)	0.5640	0.2822	1.9988		0.0558
21.7382 (16% upper)	0.8032	0.3349	2.3982		**0.0229**	0.7147 (16% upper)	1.0537	0.3545	3.3507		**0.0024**
AMA						VO_2 peak_					
Constant	5.2699	4.4164	1.1933	0.7243	0.2421	Constant	2.8305	2.9173	0.9702	0.7331	0.3397
PA (X)	−1.4680	1.7956	−0.8176		0.4201	PA (X)	−0.3419	1.1745	−0.2911		0.7730
AMA (W)	−0.1830	0.2139	−0.8554		0.3991	VO_2 peak_ (W)	−0.0414	0.0522	−0.7923		0.4344
AP*AMA (X*W)	0.1131	0.0911	1.2423		0.2238	AF*VO_2 peak_ (X*W)	0.0234	0.0240	0.9774		0.3362
Conditional effects—AMA (W)						Conditional effects—VO_2 peak_ (W)					
16.9574 (16% lower)	0.4507	0.3390	1.3296		0.1937	40.8711 (16% inferior)	0.6153	0.2908	2.1160		**0.0428**
19.3250 (64% middle)	0.7186	0.2457	2.9242		**0.0065**	48.1041 (64% mediano)	0.7847	0.2348	3.3414		**0.0022**
22.2436 (16% upper)	1.0488	0.3481	3.0130		**0.0052**	56.9272 (16% superior)	0.9913	0.3172	3.1252		**0.0039**

*Note*: Bold values indicate significant *p* values.

Abbreviations: AMA, arm muscle area; BFM, body fat mass; PA, physical activity; VO_2 peak_, peak oxygen uptake.

## 4 DISCUSSION

The main finding of this study was the direct association between PA level and PhA even after adjustment for potential confounders (age, sex, sexual maturation, viral load, CD4+ T lymphocyte count, and ART regimen). This relationship explained 31.7% of the variance in PhA among girls and 37.4% among boys. Despite this association, no mediating effect was identified in any of the four mediation models tested. A possible explanation for this result is the limited statistical power due to the sample size, which may have reduced sensitivity to detect small or moderate mediation effects. Regarding moderation effects, none of the four physical fitness variables demonstrated a significant interaction with PA in explaining PhA. However, the results suggest that both %BF and VO_2 peak_ partially moderated the relationship between PA and PhA at all evaluated levels. In contrast, variables commonly associated with sarcopenia, such as low muscle strength and reduced AMA, did not show significant interaction effects in this relationship.

Notably, the sex‐stratified analysis revealed that boys demonstrated superior physical performance indicators, including higher levels of muscular strength, aerobic capacity, and AMA (Docherty & Gaul, 1991). Girls, in turn, showed higher values of electrical resistance and a tendency toward a higher %BF, which may reflect differences in body composition that influence physical function (Marta et al., 2012). These findings are consistent with literature indicating that biological sex influences physical fitness, with boys generally presenting greater muscle mass and aerobic capacity, while girls tend to have higher fat accumulation, which can impact their physical activity levels and performance. For example, boys typically perform better in cardiorespiratory endurance tests, such as the 20‐m shuttle run, and have a higher prevalence of healthy cardiorespiratory fitness (Jones et al., 2000; Marta et al., 2012; Tomkinson et al., 2017). Furthermore, the effects of HIV and prolonged exposure to ART on physical development may vary between sexes during adolescence, reinforcing the importance of sex‐specific strategies in the assessment and promotion of physical activity and nutritional support.

Previous studies have shown that ALHIV tend to have lower overall PA scores compared to their healthy peers (Martins et al., 2017). PA plays a critical role in overall health, particularly among individuals with HIV, as it contributes to improved physical fitness and may reduce the risk of cardiovascular diseases associated with both ART and the infection itself (Chirindza et al., 2022). PhA has been linked to aerobic fitness and body composition, suggesting that individuals with HIV often present with altered hydration status and reduced body cell mass (Langer, da Costa, et al., 2020). Additionally, physically active individuals exhibit greater anabolic hormonal signaling, particularly through increased secretion of IGF‐1, insulin, and testosterone, which supports protein synthesis and the development of active cell mass (Wibawa et al., 2021). This process is also associated with increased retention of electrolytes and intracellular water.

From a physiological standpoint, regular PA promotes important adaptations in the context of HIV, such as the stimulation of mitochondrial biogenesis and the improvement of oxidative phosphorylation efficiency, which are often impaired in individuals using ART (Abrego‐Guandique et al., 2025; Maagaard & Kvale, 2009). These changes increase cellular energy production, reduce reactive oxygen species, and preserve cell membrane integrity, reflected in higher PhA values (Schwenk et al., 2000) Conversely, low PhA values may indicate reduced body cell mass, expansion of extracellular water, and unfavorable body composition, commonly observed in chronic inflammatory conditions such as HIV (Rosa et al., 2025). Exercise, associated with modulation of cytokine levels, including an increase in interleukin‐10 and a decrease in tumor necrosis factor‐alpha and interleukin‐6 (Petersen & Pedersen, 2005), helps maintain intracellular fluid balance and support muscle protein synthesis (Campa et al., 2023). These processes are particularly relevant during adolescence, a period of intense growth, in which reduced PhA values may signal lower physical fitness, compromised nutritional status, and a higher risk of adverse clinical outcomes (Rosa et al., 2025).

Simultaneously, increased lipid metabolism contributes to the reduction of chronic inflammation and enhances cell membrane fluidity, thereby facilitating ion exchange and optimizing tissue conductivity (Ayrapetyan, 2017). These physiological adaptations improve reactance and, consequently, PhA, reflecting better cellular integrity and function. Although the direct association between PA levels and PhA in adolescents living with HIV remains understudied, it is reasonable to infer that regular PA may positively influence both body composition and cellular health. This effect likely occurs through the ability of an active lifestyle to promote improvements in physical fitness components (de Araújo & de Araújo, 2000; Silva et al., 2022; Sun et al., 2024). Furthermore, regular PA has been shown to exert antioxidant effects at the mitochondrial level in individuals with HIV, which may enhance aerobic capacity and, consequently, cellular function (Valle & Hernandez, 2013).

The persistence of a direct and significant association between PA and PhA, even after adjusting for covariates, suggests that this relationship is independent of factors such as age, sex, sexual maturation, viral load, immune status, and type of ART. This finding reinforces the importance of an active lifestyle for maintaining cellular integrity and overall health, particularly in vulnerable populations such as adolescents living with HIV. Moreover, the results suggest that PA may serve as an effective strategy to enhance body composition and cellular function, regardless of clinical or demographic variables (Chirindza et al., 2022; Martins et al., 2017).

The analysis revealed that no mediating effect was present between PA and PhA according to Andrew Hayes' criteria (Hayes, 2022), indicating that the tested variables did not significantly explain the mediation model. However, partial moderation was observed for %BF and VO_2 peak_, suggesting that these factors influence the strength of the relationship between PA and PhA. In contrast, variables commonly associated with sarcopenia, such as low muscular strength and AMA, did not demonstrate a significant moderating effect. These findings suggest that, under these conditions, these variables may not significantly alter the association between PA and PhA.

Conditional effects analyses demonstrated that VO_2 peak_ partially moderated the relationship between PhA and PA levels at low, intermediate, and high values. This finding is consistent with previous literature showing a direct association between PhA and aerobic fitness in both healthy children and adolescents (Langer, da Costa, et al., 2020; Langer, de Fatima Guimarães, et al., 2020), as well as in those with chronic conditions—mainly HIV (Martins et al., 2022), regardless of the specific protocol used to assess aerobic capacity (e.g., 20‐m shuttle run, submaximal incremental ergometer test, or maximal treadmill test) (Martins et al., 2022). Additionally, both aerobic fitness and PA levels are inversely associated with %BF (de Lima et al., 2019). However, while some studies have reported a link between aerobic capacity and PhA, the nature of this relationship remains only partially established. Regarding muscular strength, significant moderating effects were observed only at low levels. These results align with findings from a systematic review (Player et al., 2019), which reported an inverse association between PhA and sarcopenia. Sarcopenia is characterized by the progressive loss of muscle mass, strength, and function, and may occur in older adults or individuals with chronic conditions such as metabolic syndrome, cardiovascular disease, HIV, or cancer (Player et al., 2019).

A considerable proportion of ALHIV in this study exhibited PhA values below 5.0°, indicating significant impairment in cellular mass and membrane integrity (Martins et al., 2020; Norman et al., 2012). This finding is indicative of a catabolic state commonly associated with HIV, characterized by muscle mass loss and deterioration of cellular function—conditions often exacerbated by chronic inflammation and malnutrition in this population (Osuna‐Padilla et al., 2022; Ott et al., 1993). A reduced PhA may reflect an elevated inflammatory response and inadequate nutrient absorption, resulting in increased vulnerability to infections and other complications (Barlow‐Mosha et al., 2013).

The low levels of PA observed among ALHIV in this study are consistent with previous findings in the literature (Chirindza et al., 2022; da Silva & Andaki, 2020; Willig et al., 2020). Several specific factors may contribute to this, including increased mitochondrial toxicity caused by chronic inflammation and ART, which can lead to excessive fatigue and reduced PA levels (Filler et al., 2014; Maagaard & Kvale, 2009). Additionally, a growing process of impoverishment disproportionately affects socioeconomically, demographically, and ethnically vulnerable groups (Atlas Brasil, 2022; Brasil, 2024). As a result, social factors, such as stigma, and psychological factors, such as depression, also negatively impact the lifestyle and PA engagement of people living with HIV (Vanable et al., 2006).

Approximately one‐third of the participants in the present study, 14 individuals (30.44%) were immunosuppressed, 12 (26.09%) with moderate immunosuppression and only 2 (4.35%) with severe immunosuppression. Chronic inflammation is a known factor associated with PhA (Stobäus et al., 2012), and PA may play a modulatory role in the inflammatory response (Gleeson et al., 2011; Gopalan et al., 2019), contributing to improved cellular membrane integrity and functionality, changes in intracellular composition, and enhanced tissue capacity (Mundstock et al., 2019). Furthermore, in line with our findings, a previous study conducted with healthy participants reported a direct association between PhA and PA levels measured by accelerometry and pedometry. These results suggest that higher PA levels are associated with greater PhA, regardless of health status (Yamada et al., 2022).

Several considerations should be noted when interpreting these results. The cross‐sectional design prevents causal inferences and may involve reverse causation. The sample, recruited from a public state‐level reference outpatient clinic, may limit generalizability. The sample size was below planned, possibly reducing the statistical power to detect mediation or moderation effects. Conceptual overlap between predictors and mediators or moderators may also have contributed to the lack of significant effects. PA assessed by self‐report is subject to recall bias and social desirability, and arm muscle area estimation may be affected by lipodystrophy‐related alterations.

On the other hand, this study presents several strengths. It assessed health indicators that are not routinely evaluated in outpatient care. As a result, reports including PA parameters, physical fitness, and cellular health (as indicated by PhA) were added to participants' medical records, along with counseling recommendations to promote PA (Dumith et al., 2021). Additionally, the study population represents a region of Brazil with a Human Development Index (HDI) below the national average (Atlas Brasil, 2022), where lifestyle‐related barriers tend to be more intense due to the limited reach of public policies. Lastly, the use of mediation and moderation analyses enabled a deeper understanding of the conditional factors influencing the relationship between PA and PhA.

## 5 CONCLUSION

The findings indicate that PA is directly associated with PhA in ALHIV, and that components of physical fitness, particularly aerobic capacity and body composition, partially moderate this relationship. These insights reinforce the importance of considering sex‐specific and fitness‐related factors when designing health promotion strategies. Moreover, future research should incorporate objective methods to measure physical activity, such as accelerometry, to enhance the accuracy and reliability of findings. Longitudinal and experimental designs are also recommended to clarify causal pathways, test the effectiveness of tailored PA interventions, and inform public health policies supported by multidisciplinary efforts and technological tools.

## AUTHOR CONTRIBUTIONS

Caio César da Silva Moura Santos was involved in conceptualization, methodology, data collection, formal analysis, writing—original draft. Luiz Rodrigo Augustemak de Lima was involved in methodology, formal analysis, writing—review and editing, and supervision. Christefany Régia Braz Costa was involved in writing—review and editing. Gustavo Gomes de Araujo, Diego Augusto Santos Silva, and Analiza Mónica Silva were involved in writing—review and editing.

## CONFLICT OF INTEREST STATEMENT

None declared.

## Supporting information


Appendix S1.

Figure S1.

Figure S2.

Figure S3.

Table S1.

Table S2.

## Data Availability

The data supporting the findings of this study are available from the corresponding author upon reasonable request. Due to ethical restrictions and the need to protect participant confidentiality.
