# Supplementary figures and images for: Associations of physical activity with phase angle in adolescents living with HIV: The moderating and mediating roles of physical fitness
Source: Physiol Rep. 2026 Feb 3;14(3):e70696. doi: 10.14814/phy2.70696 (PMC12867954; doi:10.14814/phy2.70696)

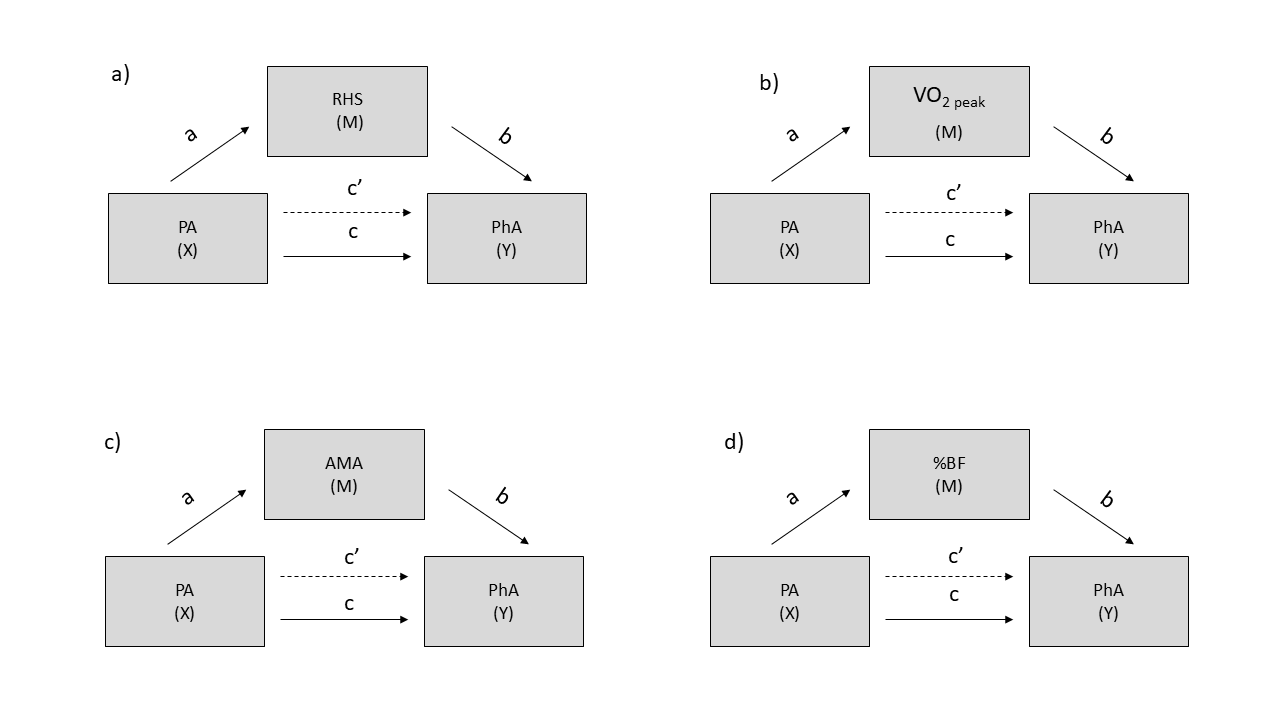

Supplement: Supplementary file 2 — Figure S1. [file PHY2-14-e70696-s004.tif]

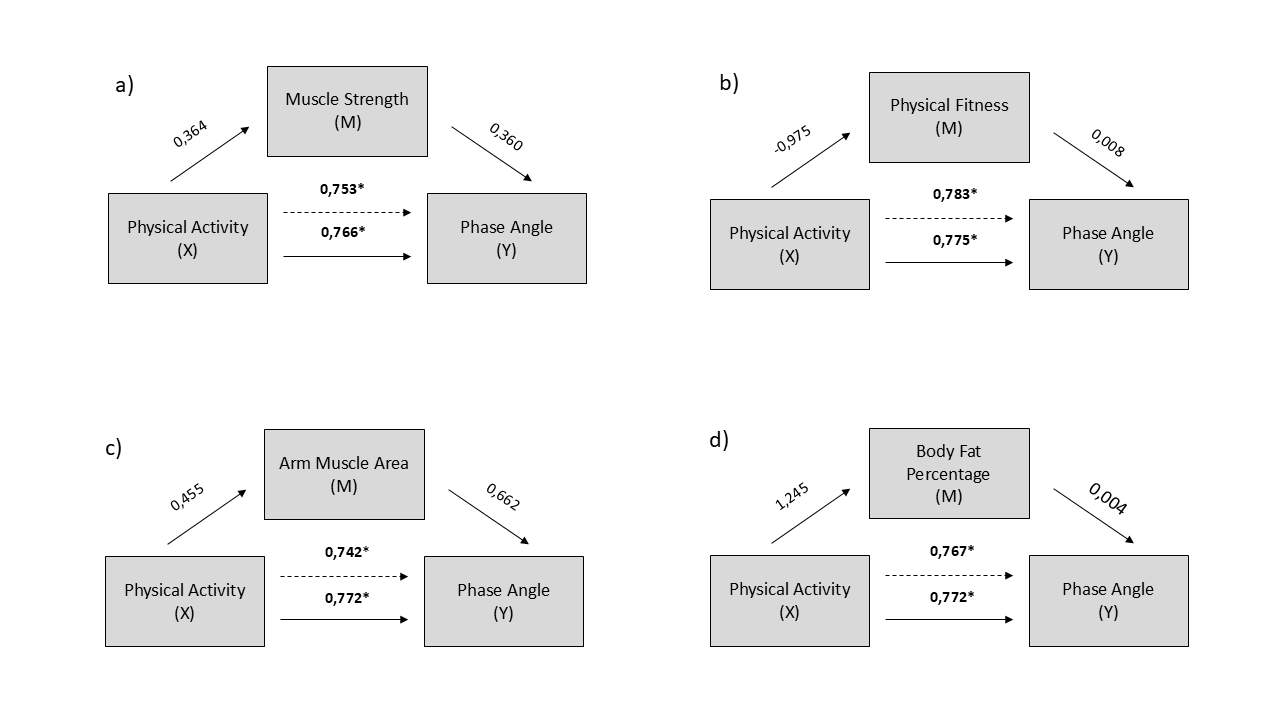

Supplement: Supplementary file 4 — Figure S3. [file PHY2-14-e70696-s001.tif]
