# Supplementary material for: Associations of physical activity with phase angle in adolescents living with HIV: The moderating and mediating roles of physical fitness
Source: Physiol Rep. 2026 Feb 3;14(3):e70696. doi: 10.14814/phy2.70696 (PMC12867954; doi:10.14814/phy2.70696)
Supplement: Supplementary file 3 — Figure S2. [file PHY2-14-e70696-s006.docx]

**Supplementary Figure 2.** Frequency of physical activities among girls (A) and boys (B) adolescents living with HIV, Brazil, 2024.
