# Supplementary material for: Associations of physical activity with phase angle in adolescents living with HIV: The moderating and mediating roles of physical fitness
Source: Physiol Rep. 2026 Feb 3;14(3):e70696. doi: 10.14814/phy2.70696 (PMC12867954; doi:10.14814/phy2.70696)
Supplement: Supplementary file 5 — Table S1. [file PHY2-14-e70696-s005.docx]

| **Supplementary table 1**. Regimen of Combined Antiretroviral Therapy of adolescents living with HIV. Brazil. 2024 | | |
| --- | --- | --- |
| nº | Girls (n = 25); 14 with protease inhibitors (in bold) | Boys (n = 22); 14 with protease inhibitors (in bold) |
| 1 | **Atazanavir**, Tenofovir, Lamivudina, **Ritonavir** | Tenofovir, **Atazanavir**, Raltegravir, **Ritonavir** |
| 2 | Tenofovir, Lamivudina, Enfavirenz | Tenofovir, Lamivudina, Efavirenz |
| 3 | Etravirina, **Ritonavir**, Tenofovir, Lamivudina, **Atazanavir** | Raltegravir, Zidovudina, Lamivudina |
| 4 | Efavirenz, Zidovudina, Lamivudina | Dolutegravir, **Ritonavir**, Tenofovir, **Atazanavir** |
| 5 | **Ritonavir**, Tenofovir, Lamivudina, **Atazanavir** | Tenofovir, Lamivudina, Efavirenz |
| 6 | Tenofovir, **Ritonavir**, Lamivudina, **Atazanavir** | **Ritonavir**, **Atazanavir**, Zidovudina, Lamivudina |
| 7 | **Ritonavir**, Tenofovir, Lamivudina, Raltegravir, **Atazanavir** | Tenofovir, Lamivudina, Enfavirenz |
| 8 | Tenofovir, **Atazanavir**, **Ritonavir** | **Ritonavir**, **Atazanavir**, Zidovudina, Lamivudina |
| 9 | Tenofovir, Lamivudina, Doluteglavir | Zidovudina, Lamivudina, **Atazanavir**, **Ritonavir** |
| 10 | Dolutegravir, Tenofovir, Lamivudina | **Ritonavir**, Tenofovir, Lamivudina, **Atazanavir** |
| 11 | **Atazanavir**, Tenofovir, Lamivudina, **Ritonavir**, Dolutegravir | **Ritonavir**, Tenofovir, Lamivudina, **Atazanavir** |
| 12 | **Ritonavir**, Tenofovir, Lamivudina, **Atazanavir** | **Ritonavir**, Raltegravir, **Atazanavir**, Tenofovir |
| 13 | Efavirenz, Zidovudina, Lamivudina | Zidovudina, Lamivudina, **Atazanavir**, Raltegravir, **Ritonavir** |
| 14 | **Ritonavir**, Dolutegravir, **Darunavir** | Tenofovir, Lamivudina, **Ritonavir**, **Atazanavir** |
| 15 | **Ritonavir**, Tenofovir, Lamivudina, **Atazanavir** | Dolutegravir, Tenofovir, Lamivudina |
| 16 | **Ritonavir**, Tenofovir, Lamivudina, **Atazanavir** | Tenofovir, Lamivudina, Dolutegravir |
| 17 | Dolutegravir, Zidovudina, Lamivudina | Tenofovir, **Atazanavir**, **Ritonavir**, Dolutegravir |
| 18 | Tenofovir, Lamivudina, Efavirenz | **Ritonavir**, Tenofovir, Lamivudina, **Atazanavir** |
| 19 | Tenofovir, Lamivudina, Enfavirenz | Tenofovir, Lamivudina, Dolutegravir |
| 20 | Enfavirenz, Tenofovir, Lamivudina | Raltegravir, Tenofovir, Lamivudina, **Ritonavir**, **Darunavir** |
| 21 | **Ritonavir**, Dolutegravir, **Darunavir** | Nevirapina, Zidovudina, Lamivudina |
| 22 | Tenofovir, Lamivudina, Enfavirenz | **Atazanavir**, Tenovir, Lamivudina, **Ritonavir**, Raltegravir |
| 23 | **Ritonavir**, Tenofovir, Lamivudina, **Atazanavir** | - |
| 24 | Dolutegravir, Tenofovir, Lamivudina | - |
| 25 | Etravirina, **Ritonavir**, **Atazanavir**, Tenofovir | - |
