# Supplementary material for: Associations of physical activity with phase angle in adolescents living with HIV: The moderating and mediating roles of physical fitness
Source: Physiol Rep. 2026 Feb 3;14(3):e70696. doi: 10.14814/phy2.70696 (PMC12867954; doi:10.14814/phy2.70696)
Supplement: Supplementary file 6 — Table S2. [file PHY2-14-e70696-s003.docx]

| **Supplementary Table 2.** Spearman Correlation of study variables and covariates with the phase angle of adolescents living with HIV. Brazil. 2024. | | | |
| --- | --- | --- | --- |
| **Variables** | **Correlation coefficient (p)** | | |
|  |  | | |
|  | **Phase Angle** | | |
|  |  | | |
|  | **Total** | **Girls** | **Boys** |
|  | **(n = 47)** | **(n = 25)** | **(n = 22)** |
|  |  |  |  |
| Age | 0.20 (0.21) | -0.02 (0.94) | **0.43 (0.05)** |
| Sexual Maturation | 0.13 (0.43) | -0.13 (0.58) | 0.35 (0.14) |
| Viral Load | -0.29 (0.06) | **-0.43 (0.05)** | 0.00 (0.99) |
| CD4+ T Lymphocytes | -0.08 (0.59) | -0.05 (0.81) | -0.25 (0.28) |
| ART time | 0.24 (0.12) | 0.22 (0.35) | 0.16 (0.49) |
| Physical Activity | **0.39 (0.01)** | **0.45 (0.04)** | 0.31 (0.17) |
| ART – antiretroviral therapy; VO_2 peak_ – Peak Oxygen Uptake; Significant correlations are in bold (p < 0.05). All correlations used Spearman’s method due to asymmetry in the “phase angle” dependent variable. | | | |
